# AlphaFold2 reveals commonalities and novelties in protein structure space for 21 model organisms

**DOI:** 10.1038/s42003-023-04488-9

**Published:** 2023-02-08

**Authors:** Nicola Bordin, Ian Sillitoe, Vamsi Nallapareddy, Clemens Rauer, Su Datt Lam, Vaishali P. Waman, Neeladri Sen, Michael Heinzinger, Maria Littmann, Stephanie Kim, Sameer Velankar, Martin Steinegger, Burkhard Rost, Christine Orengo

**Affiliations:** 1 Institute of Structural and Molecular Biology, University College London, London, WC1E 6BT UK; 2 Department of Applied Physics, Faculty of Science and Technology, Universiti Kebangsaan Malaysia, 43600 Bangi, Selangor Malaysia; 3 TUM (Technical University of Munich) Department of Informatics, Bioinformatics & Computational Biology, i12, Boltzmannstr 3, 85748 Garching/Munich, Germany; 4 School of Biological Sciences, Seoul National University, Seoul, South Korea; 5 Artificial Intelligence Institute, Seoul National University, Seoul, South Korea; 6 European Molecular Biology Laboratory, European Bioinformatics Institute, Hinxton, UK; 7 Institute for Advanced Study (TUM-IAS), Lichtenbergstr. 2a, 85748 Garching/Munich, Germany; 8 TUM School of Life Sciences Weihenstephan (WZW), Alte Akademie 8, Freising, Germany

**Keywords:** Protein structure predictions, Protein analysis

## Abstract

Deep-learning (DL) methods like DeepMind’s AlphaFold2 (AF2) have led to substantial improvements in protein structure prediction. We analyse confident AF2 models from 21 model organisms using a new classification protocol (CATH-Assign) which exploits novel DL methods for structural comparison and classification. Of ~370,000 confident models, 92% can be assigned to 3253 superfamilies in our CATH domain superfamily classification. The remaining cluster into 2367 putative novel superfamilies. Detailed manual analysis on 618 of these, having at least one human relative, reveal extremely remote homologies and further unusual features. Only 25 novel superfamilies could be confirmed. Although most models map to existing superfamilies, AF2 domains expand CATH by 67% and increases the number of unique ‘global’ folds by 36% and will provide valuable insights on structure function relationships. CATH-Assign will harness the huge expansion in structural data provided by DeepMind to rationalise evolutionary changes driving functional divergence.

## Abstract (web summary)

A new protein domain classification protocol incorporating deep learning strategies for detecting sequence and structure similarities between domain is used to systematically study and analyse the predicted AlphaFold2 structural models for proteins of 21 organisms.

## Introduction

For over 30 years, the pace of sequencing proteins has outstripped that of structure determination and at the start of 2022, the non-redundant protein sequence data in UniRef90^1^ was 1000-fold greater than the associated 3D data. Methods of protein structure prediction have progressed in the same time-frame. Especially for sequences having a close homologue (>40% sequence identity) of known 3D structure, homology modelling can provide an accurate structure for many biological analyses^2–4^. However, there still remains a significant deficit, including for human proteins linked to disease^5^. Even for many of the well-studied prokaryotic model organisms (e.g., *Escherichia coli*, *Bacillus subtilis*), the proportion of the proteome (protein coding part of genome) for which high-resolution protein 3D structures have been experimentally determined or can be reliably predicted remains below 55%. The structural coverage is lower for eukaryotic model organisms, e.g., only 36% of human and 30% of baker’s yeast proteins are, at least, partially covered by structures^6^.

Over the last ten years, a number of developments in template-free ab-initio structure prediction (e.g., co-variation^7^, deep learning from vast sequence data^8–11^) have led to promising improvements, and recently the AlphaFold2 (AF2) method developed by DeepMind has reached an impressive level of performance as evidenced by independent assessment^12,13^.

In August 2021, in collaboration with PDBe at EMBL-EBI, DeepMind provided via AlphaFold DB v1 AF2 3D-models for 21 selected model organisms (including human, mouse, *Arabidopsis thaliana*, rice, yeast and *E. coli*), comprising 365,184 model structures altogether^14^. Information on global and per residue model accuracy is provided for each model. The scale and accuracy of this modelling initiative is likely to be a gamechanger for biological and medical research as protein structure data is key to understanding the molecular mechanisms of proteins and the impact of genetic variations on their molecular function and, therefore the biological processes they participate in.

To exploit these data, it would be valuable to assign the modelled domains to their evolutionary families to better understand how genetic variations modify structure and ultimately function. Proteins comprise combinations of domains, and millions of combinations of domains exist across genomes^15,16^. Since the protein domain is thought to be an important module contributing specific functional features, a tractable approach for handling the vast number of proteins in nature is to organise by domain family. Currently, most experimentally characterised domain structures have been assigned to fewer than 6000 evolutionary families^17,18^.

Over the last 25 years, several domain-based protein structure classifications have emerged (SCOP^19^, CATH^18^, SCOPe^20^, SCOP2^21^, ECOD^17^) which assign experimental structures of proteins from the Protein Data Bank (PDB) to evolutionary superfamilies. ECOD, SCOPe, and CATH are the most comprehensive, classifying 90% or more of PDB. Since the advent of large-scale genome sequencing, the CATH classification has also identified homologous domain sequences for CATH superfamilies in UniProt^22^ and complete genomes from Ensembl^23^. This expands the sequence population of CATH superfamilies by nearly 300-fold and brings in more functional annotations for the proteins^24^.

The provision of high quality AF2 structural models from DeepMind for 21 organisms gives an opportunity to significantly expand the structural data in CATH superfamilies. This would allow us to provide multiple structural alignments and identify structurally conserved features correlating with functional motifs. It would also allow us to assess how representative existing structural superfamilies are of domains in nature and to reveal novel folding architectures and motifs not in the PDB.

Here we present a new classification protocol CATH-Assign, which incorporates novel and rapid deep learning strategies for detecting sequence and structure similarities between domains to rapidly classify structures. We applied the protocol to analyse protein structures of 21 model organisms predicted by AF2. We removed models deemed to be low quality by the AF2 developers and any that had long stretches of residues or a large proportion of residues with no secondary structure assignment. We also removed models in which the overall contact density of residues was unusual compared to distributions seen in the PDB. Of the nearly 700,000 domains provided by AF2, only 52% of models met these criteria (369,512 domains) but 92% of them could be assigned to 3253 existing CATH superfamilies. The remaining domains could be clustered into 2367 putative new superfamilies. Manual curation of very remote homologues is extremely time-consuming but for a subset of these novel superfamilies, comprising human models, we manually identified 25 likely new superfamilies. By bringing the AF2 models into CATH we expand the number of ‘global fold’ groups (in which structural relatives in superfamilies superimpose well) by 36%. Furthermore, we increase the number of functional families, CATH-FunFams (in which relatives are predicted to have high structural and functional similarity and for which we have at least one relative with experimental characterisation reported in the Gene Ontology) from 8 to 30%. This suggests that the release of a further 214 million models by DeepMind will bring significant insights on how structures (and, therefore their functions) diverge during evolution. The data is available grouped by CATH Superfamily and by organism through the 3D-Beacons network^25^, Zenodo (10.5281/zenodo.7404988, https://zenodo.org/record/7404988), and the CATH FTP (ftp://orengoftp.biochem.ucl.ac.uk/alphafold/cath-v4.3.0-model-organisms).

## Results

### Proportion of AlphaFold2 models that can be brought into CATH

#### Identification of domains in AF2 protein models

**Fig. 1:**
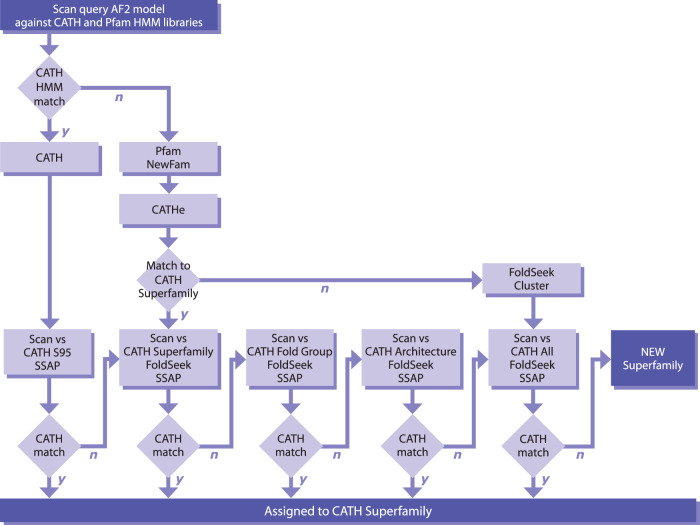
Overview of the CATH-Assign protocol used to process the predicted AF2 domains. CATH-HMM (labelled as CATH) are structurally compared against the Superfamily non-redundant representative that they match. Pfam and NewFam domains are classified into CATH Superfamilies using the CATHe predictor where possible. A cascade method is used to validate, starting with structure scans against non-redundant domains in the CATHe predicted Superfamily, then the predicted Topology, Architecture, and finally domains from all superfamilies if necessary.

We applied an in-house Hidden-Markov Model-based protocol CATH-Resolve-Hits (CRH)^26^ to assign domain regions in all sequences from the 21 model organisms modelled by AlphaFold2 (AF2) (see “Methods” for description and Fig. 1). CRH identifies domains that can be putatively assigned to either CATH superfamilies, structurally uncharacterised Pfam families or novel superfamilies (NewFams). We include structurally uncharacterised Pfam families to improve domain boundary resolution as Pfam families are manually curated and of high quality.

Using CRH and CATH (version 4.3), we assigned each region in the UniProt sequences of all AF2 models to four possible categories: (1) CATH-experimental (CATH-PDB) if there is a structure for this domain in the PDB and predicted domains comprising (2) CATH-HMM, (3) Pfam or (4) NewFam assignments, for which there is no relative in CATH or Pfam. We subsequently chopped these structural regions from the corresponding AF2 protein 3D-model (See Fig. 1 for the workflow and Supplementary Fig. 1 for the numbers of domains in each category).

**Fig. 2:**
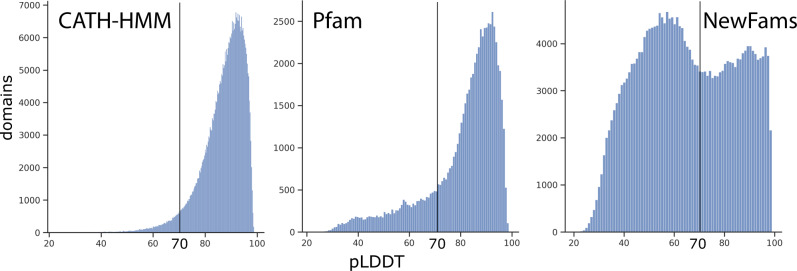
Average model quality. The plots show the distribution of average pLDDT scores for domains divided by source. The pLDDT threshold for confident model quality is highlighted (≥70).

AF2 CATH-HMM models tend to be higher quality than structurally uncharacterised Pfam or NewFam domains (see Fig. 2 and Supplementary Fig. 2 for more details). This is likely because AF2 provides better quality models if the structure or close homologue is present in the PDB^27^. CATH-PDB and CATH-HMM AF2 models also have higher percentages of ordered residues (i.e., with secondary structure assignment (see Fig. 2 and Supplementary Fig. 2 for more details)) than the other domain types. Since AF2 model quality relies on query alignment depth, the low quality for NewFam domains could suggest large levels of disorder or very shallow alignments for these families, with few sequences or species in them^5^.

### Removing models with poor quality or problematic features

Only confident domain models (as described below) were considered for assignment to CATH superfamilies. Models that contain problematic features are likely to make it difficult to recognise a structural relationship. We used a number of criteria, including the threshold given by AF2 developers for confident model quality (i.e., predicted local distance difference (pLDDT) ≥ 70)^12^. We also removed models that had long regions of residues (>30% of the domain) with no secondary structure assignment or where a large proportion of the domain (>65%) comprised residues with no secondary structure assignment (see “Methods”). We also imposed the criteria used for the classification of domains into CATH, that domains have ≥40 residues and ≥3 secondary structure elements. Finally, we removed models in which the average contact density of the residues was very unusual compared to the values found for experimentally characterised protein domains in the PDB (see Supplementary Tables 1 and 2, Supplementary Fig. 3 and “Methods” for more details on methods used to filter the AF2 domain models and for the numbers of models removed and remaining for each type). Using these criteria, we removed 339,428 domains from further analyses.

### Proportion of domains that can be assigned to CATH superfamilies

#### Analysis of CATH-HMM predicted domains

For 273,346 domains with clear homologues in CATH (i.e., domains matching HMMs built on CATH structural representatives), we compared the domain to the structural representative used to seed the HMM. We used our slow but sensitive SSAP method to do this (see Supplementary for more details). On average, the SSAP score for the CATH-HMM domains aligned over the non-redundant (S95) representative was 84.8, with an average overlap over the S95 representative of 81.5%. A total of 246,143 CATH-HMM domains could be classified into CATH superfamilies.

### Analysis of Pfam and NewFam predicted domains

To determine whether these domains were distant homologues of CATH superfamilies, we used a recently established protein Language Model (pLM), Prot-T5^28^, to generate protein sequence embeddings of the domains. We then used CATHe^29^, an established artificial neural network (ANN) predictor trained on known CATH superfamily domain embeddings (see Methods), to predict CATH superfamilies for these uncharacterised domains. In previous studies, CATHe showed 86% accuracy on all CATH superfamilies and 98% for prediction of the 50 most highly populated superfamilies that currently account for 40% of CATH domains^29^. 43.4% of Pfam domains and 23.8% of NewFam domains could be assigned to CATH superfamilies with high confidence by CATHe. We then validated the predicted domains by performing structure comparison against all non-redundant (S95) relatives in the predicted CATH superfamily.

Some superfamilies are very large (sizes of the matched superfamilies ranged from 1 to 4546 non-redundant relatives). We therefore applied a new structure comparison method, Foldseek, which is several orders of magnitude faster than the well-established TM-align method while matching its sensitivity^30,31^. We benchmarked this method using manually curated CATH domain assignments to determine an acceptable threshold for superfamily assignment (see Supplementary Materials, section Foldseek benchmark, Supplementary Figs. 13 and 14).

A cascading approach was used, i.e., if no match was obtained using Foldseek, our in-house SSAP method^32^ was used, which is very much slower but slightly more sensitive than Foldseek (See Supplementary Fig. 4). Suitable SSAP thresholds were also benchmarked (see Supplementary Materials, section SSAP Benchmark, Supplementary Figs. 11 and 12). Using these approaches a total of 37,603 Pfam and NewFam domains could be classified into CATH superfamilies.

### All domains unmatched to CATH superfamilies by CATH-HMM, CATHe, Foldseek, SSAP

Finally, all unmatched domains were scanned with Foldseek and SSAP against AF2 domains assigned to CATH superfamilies by the steps above. This brought a further 43,646 domains into CATH superfamilies.

**Fig. 3:**
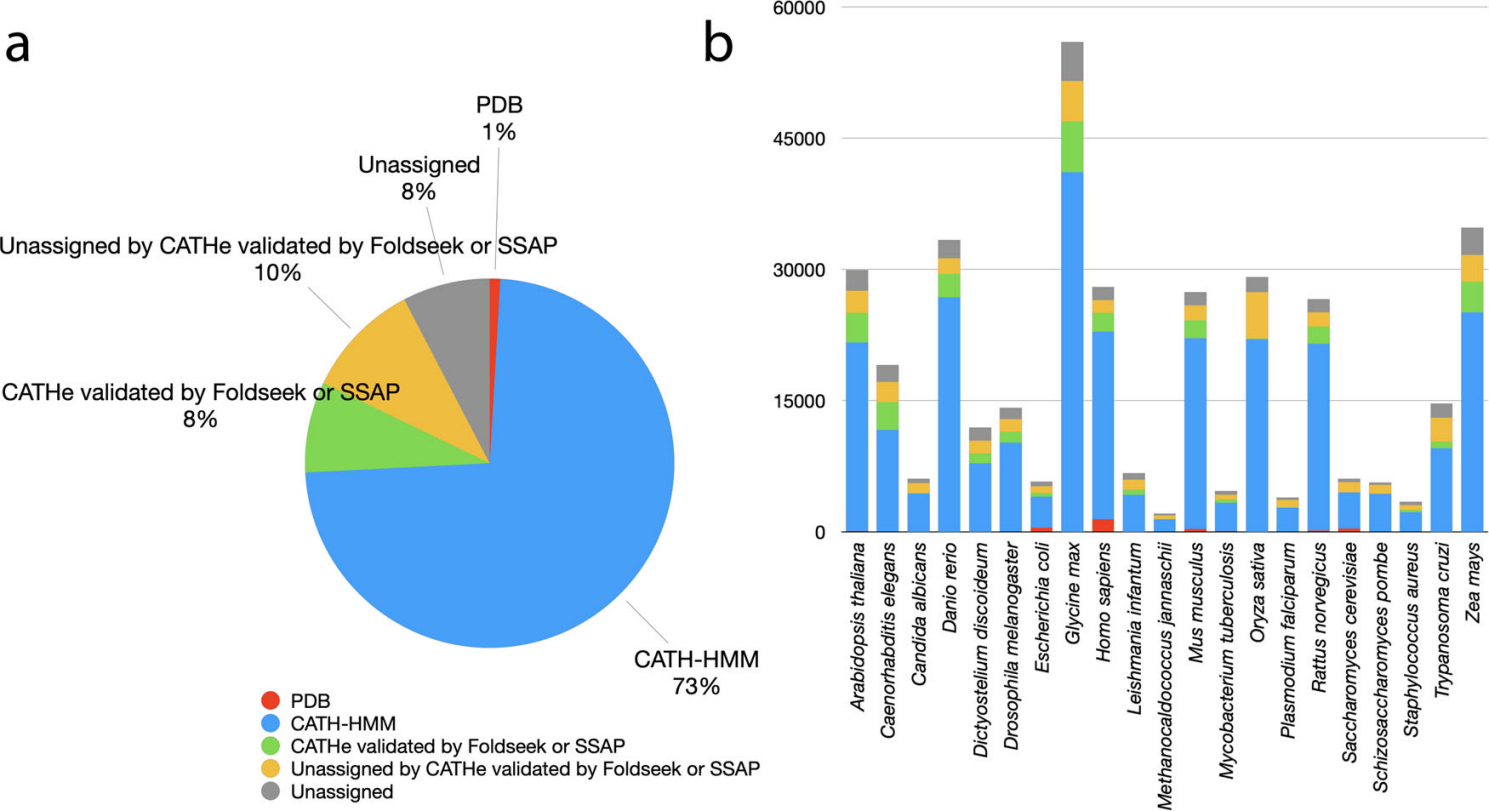
CATH coverage of the AlphaFold2 dataset. **a** Overview of domain quality and ontology for the total AlphaFold2 dataset and **b** subdivided by each proteome.

In summary, of the 369,512 domains passing our selection criteria and analysed using our sequence- (CATH-HMM, CATHe) and structure-based (Foldseek, SSAP) protocols, 341,213 (92.3%) could be assigned to CATH superfamilies, representing a 67% expansion in CATH domains (341,213/500,238 = 0.67). A majority of these domains (79.3%) were relatively close homologues that could be detected by HMM based strategies (Fig. 3). Table 1 shows the numbers of domains assigned to CATH superfamilies and the resulting percentage expansion in CATH.

**Table 1:** Number of domains at each processing step by source.

	(A) Total number of domains	(B) Confident domains (% of A)	(C) Confident domains with CATHe assignments (% of B)	Confident structurally validated by CATH-PDB (% of B)	Confident structurally validated by CATH-expanded (% of B)
CATH-PDB	3407	3407	----	3407 (100%)	0 (0%)
CATH-HMM	387492	273346 (70.5%)	----	256557 (93.9%)	12403 (4.5%)
Pfam	87681	38046 (43.4%)	34110 (89.7%)	13738 (36.1%)	8412 (22%)
NewFam	230360	41892 (18%)	12154 (29%)	4645 (11.1%)	2649 (6.3%)

**Fig. 4:**
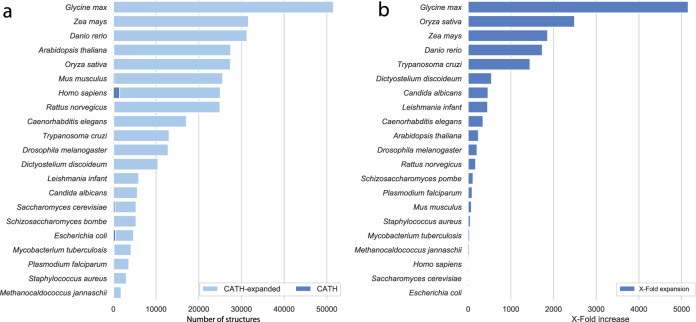
Structural coverage expansion. **a** Expansion in structural coverage by total number of structural domains and **b** fold-wise by validated CATH-HMM, Pfam and NewFams domains models for the 21 organisms in the AlphaFold2 dataset.

These validated structures represent an average increase in structural coverage by CATH domains of 742-fold over the 21 model organisms (see Fig. 4). The expanded coverage is particularly evident in the case of *Glycine max* and *Oryza sativa*, with a 5100-fold and 2400-fold increase in structural coverage of domains belonging to these organisms.

Considering the class of the domains, 29.8% map to mainly-alpha superfamilies, 23.4% to mainly-beta and 46.7% to alpha-beta, and these proportions are quite similar to those observed for experimental domain structures in CATH, albeit with a slightly lower percentage of alpha superfamilies in CATH experimental (alpha: 21.2%, beta: 25.3%, alpha/beta: 53.5%). This overabundance of mainly-alpha superfamilies has been noticed also in other AF2 domain classification efforts based on SCOPe^20^. Supplementary Fig. 5 shows the average expansion of each CATH architecture. The top 200 most highly populated superfamilies in CATH (sometimes referred to as MegaFamilies) comprise nearly 70% of the experimental structures in CATH. A large portion of AF2 domains (62%) map to these superfamilies.

### Expansion of functional families by AlphaFold2 structural data

**Fig. 5:**
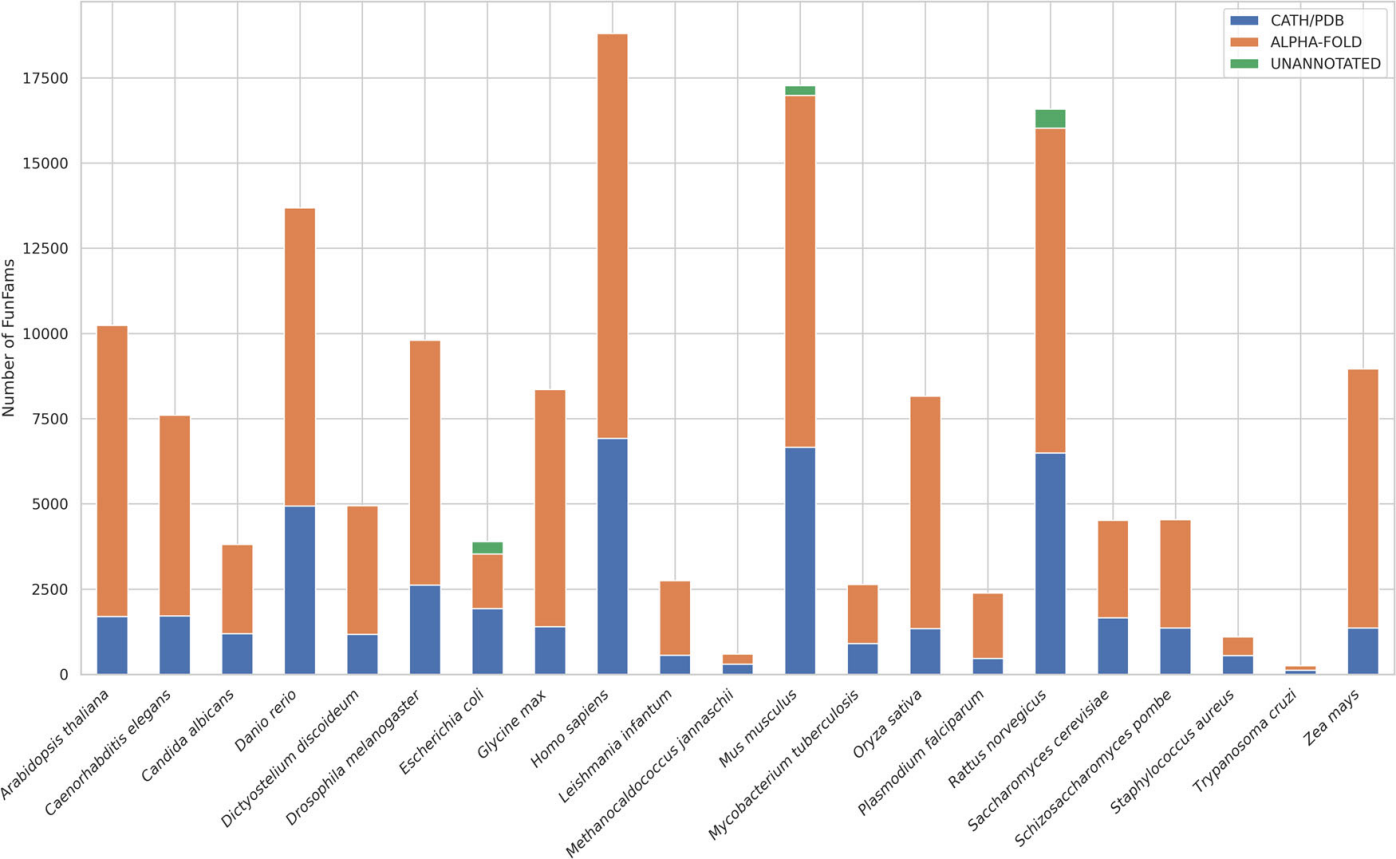
Structural coverage expansion of CATH FunFams by AlphaFold domains. Initial coverage by CATH/PDB (blue), additional coverage by AlphaFold2 models (orange) and unannotated (green).

Each CATH superfamily has been classified into functional families (FunFams), in which relatives are predicted to have similar structures and functions^24,33,34^. Since this sub-classification is computationally expensive^34^, FunFams have only been generated for families in which at least one relative has an experimental characterisation in the Gene Ontology. Version 4.3 of CATH contains 212,872 FunFams comprised of 322,202 domains (i.e., 64% of all CATH domains)^35^. Only 17,208 FunFams (8%) have at least one domain which has been structurally characterised. Assignment of AF2 3D-models into CATH superfamilies by the protocol described above (Fig. 1) brought 104,306 more structural relatives into the FunFams. This increased the proportion of FunFams that have at least one relative with an experimental or AF2 structure by 3.7-fold overall (from 8 to 30%) and by up to 6.6-fold, depending on the organism (see Fig. 5). Supplementary Fig. 6 shows that FunFams with higher numbers of relatives and species within them were more likely to have AF2 models predicted with confidence.

**Fig. 6:**
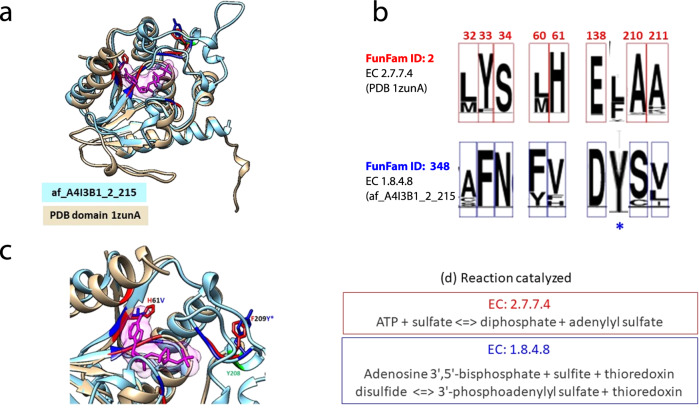
Functional diversity between protein families revealed using Alphafold2: The HUP superfamily as an example. Members of HUP superfamily (CATH ID:3.40.50.620) possess a common structural core comprising a Rossmann αβα-sandwich fold. Several functional families within the HUP superfamily lacking a representative PDB structure now possess a representative domain from Alphafold2 enabling characterisation of putative functional sites in their associated functional families. For example, the Phosphoadenosine phosphosulfate reductase-like protein family (PPR) (CATH FunFam ID: FunFam-348; EC: 1.8.4.8) has a high-quality AF2 domain available for the poorly studied PPR protein from L*eishmania infantum* (af_A4I3B1_2_215; pLDDT: 94.87). This protein has no close homologue in the PDB (>30% sequence identity). We compared the AF2 model with the representative PDB structure (1zunA) from its closest Functional Family in CATH i.e., Sulphate adenylyltransferase family (SAT) [FunFam-02, representative PDB:1zunA; EC:2.7.7.4]. **a** Superposition of structure representatives from FunFam-348-PPR and FunFam-02-SAT. Residues conserved in both families are coloured green, FunFam-specific residues, blue (af_A4I3B1_2_215, for FunFam-348-PPR) and red (1zunA, for FunFam-02-SAT). **b** FunFam-2-SAT is involved in ATP hydrolysis, an essential process for its function. Analysis of conserved residues using Scorecons indicated that most active site residues (shown in red) are conserved differently between the two functional families. Moreover, there is a change in catalytic residue site (indicated as blue *) in PPR i.e., F(/L)209Y. The height of each residue indicates its degree of conservation. **c** Close-up view of differentially conserved positions between the families in the active site FunFam-02-SAT (red) and FunFam-348-PPR (blue). The substrate molecule (AGS) in FunFam-02-SAT (1zunA) is shown in magenta. Chemically different residues highlighted in the substrate-binding site (H61V) and catalytic site (F174Y) of FunFam-348. **d** Catalytic mechanisms for the two Enzyme families.

The increase in structural characterisation of FunFams by AlphaFold2 models will provide valuable insights into how sequence and structural modifications drive functional changes across superfamilies, by facilitating the identification of putative functional sites and surfaces. For example, in the structurally and functionally diverse HUP superfamily (3.40.50.620) we used the structural representatives provided by AF2 to examine changes in highly conserved residues located in the active site pocket between two FunFams (Phosphoadenosine phosphosulfate reductase-like protein family (PPR) and Sulphate adenylyltransferase family SAT)), one of which has experimental structural characterisation of the binding pocket (Fig. 6). The AF2 3D-model allows us to compare the location of highly and differentially conserved residues between the two FunFams in the binding pocket, which contributes to understanding the mechanisms that drive changes in function between these two groups of relatives (Fig. 6).

### Identification of new superfamilies and new folds

To assess whether the remaining 27,112 unclassified AF2 domains belonged to novel superfamilies, we compared the domains against each other first using Foldseek and then SSAP, and structurally similar domains were clustered together (see Methods for more details), giving 4235 structural clusters. Of these, 1154 clusters had representatives that matched structures in the PDB not yet classified in CATH. A further 714 were clearly multidomain as we found using Foldseek that part of the region structurally matched a domain in CATH-expanded. For the remaining 2367 detailed manual analysis is required to check for very remote homology to the closest matched CATH superfamily relatives^36^. This is time consuming as it requires extensive visual inspection and checking information in the literature. Furthermore, since we had observed that bringing AF2 domain models into CATH improved the detection of remote homologues by providing greater coverage of the family (see section “Proportion of domains that can be assigned to CATH superfamilies”), we reasoned that detection of very remote homologues would be better enabled once further AF2 domain models from the AlphaFold UniProt release of 214 million proteins had been processed and brought into CATH.

We, therefore, opted to manually examine the 618 putative new families/folds containing at least one relative from *Homo sapiens*, to assess the nature of new families and folds and assess whether there were any interesting and unusual structural features or perhaps previously missed problematic features that could explain why they had not been matched to existing CATH superfamilies/folds. We examined a representative from each of the 618 structural clusters.

**Fig. 7:**
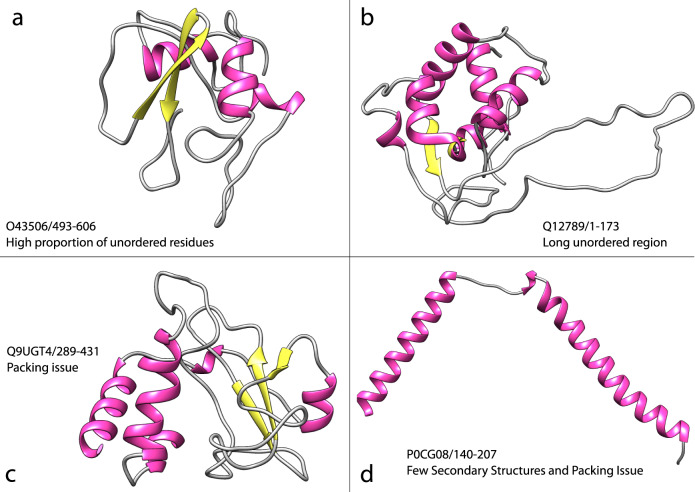
Issues encountered when processing domains not assigned to CATH. Each structure figure was generated using UCSF Chimera^36^, with identifiers in the format UniProt_ID/start-stop. Examples of poor models **a** High proportion of unordered residues. **b** Presence of long unordered regions. **c** Residue packing problems. **d** Less than three secondary structures and packing problems.

For 89% of the clusters, we found possible explanations for the lack of structural matches with domains in CATH superfamilies. Some of these cases (42%) appear to be regions comprising more than one domain (see Fig. 7c). This was most frequent for domains assigned to structurally uncharacterised Pfam families, for which the lack of structure can make it difficult to determine domain boundaries. A further 23% appeared to contain problematic regions e.g., large unstructured regions at either termini or poorly packed secondary structures (see Fig. 7a, b and d) not picked up by the thresholds on our filtering programmes, but which would make it hard to recognise structural similarities with relatives in CATH. Some of these features may reflect inaccurate models caused by the small family sizes available to AF2 to model the structures. To confirm the novelty of these putative novel folds, we performed a final check by scanning the remaining 26 cluster representatives using TM-align against all CATH S95 representatives, after benchmarking it on our benchmark dataset (see Supplementary section TM-align Benchmark, Supplementary Figs. 15 and 16). Using TM-align we were able to assign a further cluster (Q9P6K6/1-137) to a CATH superfamily, thus reducing the number of putative new folds to 25.

**Fig. 8:**
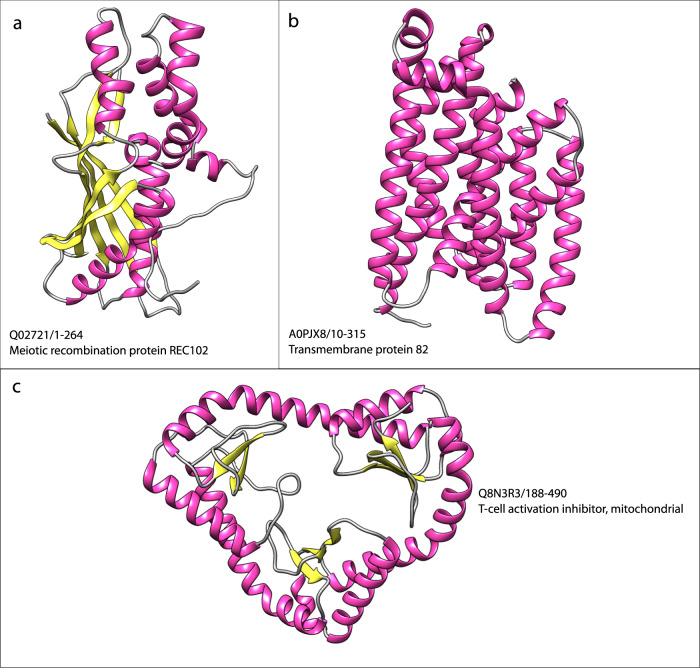
New Structural Superfamilies. Each structure figure was generated using UCSF Chimera^36^, with identifiers in the format UniProt_ID/start-stop. **a** Meiotic recombination protein REC102. **b** Transmembrane protein 82. **c** T-cell activation inhibitor, mitochondrial.

There were 25 putative clusters remaining that could not be assigned to a CATH superfamily and did not have any problematic features as discussed above. These represent putative new superfamilies (see Supplementary Figs. 7 and 8 for all the novel structural superfamilies. A selection of 3 are shown in Fig. 8 below). Some looked similar to known families in CATH (Fig. 8a, b) and it is likely that with further processing of the AFDB release of UniProt and subsequent expansion in the coverage of CATH with these models, these putative novel domains will match a CATH AF2 relative. Some have unusual structural architectures. For example, there is a heart shaped arrangement (Fig. 8c) that may comprise three small repeat domains (small alpha beta 2-layer domains) linked by longer helices although it is hard to see how individual domains could easily be carved out of this. This protein, whose existence was confirmed also by RosettaFold^37^, is a mitochondrial T-cell activation inhibitor involved in T-cell activation and memory formation and we found other related AF2 domain structures in 14 species. We were also able to build a dimer with AlphaFold Multimer^38^ and in complex with two interactors (CLIC3, MAN1A1) predicted by STRING^39^. Recent cryo-EM structures deposited in PDB indicates that this protein forms an open conformation when in complex (as shown in *T.brucei* as part of the mitochrondrial assembleosome^40,41^), with residues in region 450–500 unfolding in a ‘open-heart’ conformation.

We will revisit these putative novel human superfamilies and those from the other model organisms once we have processed further domains from the latest release of AlphaFold Database, containing over 214 million predicted structures covering the entirety of UniProt.

**Fig. 9:**
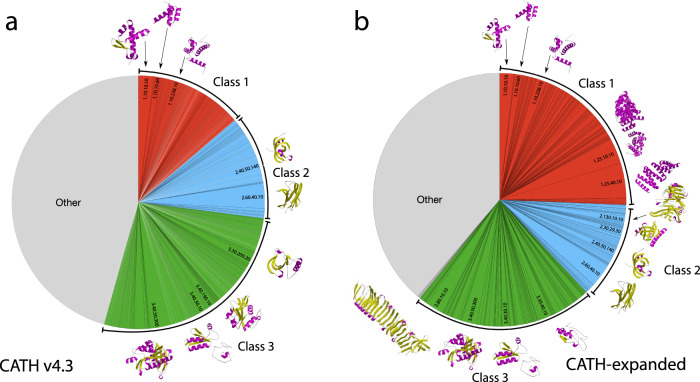
Expansion in structural diversity in CATH by predicted AlphaFold structural models. **a** Distribution of structural cluster sizes coloured by CATH class in CATH v4.3 and **b** expanded by AlphaFold structural models.

Although ~92% of the confident AF2 models can be assigned to existing structural superfamilies in CATH, they bring considerable structural novelty. CATH superfamilies are sub-clustered into groups of relatives (structurally similar groups (SSGs)) that can be superposed well. These can be considered as ‘global fold’ groups as there are distinct changes outside the common structural ‘core fold’ (see Fig. 9). Currently there are ~28,000 such global fold groups in CATH. Adding AF2 structures increases this number by ~36% to 38,000.

## Discussion

For the last 50 years, the number of structures known experimentally has been a small proportion of the numbers of protein sequences determined. Just prior to the release of DeepMind’s AlphaFold2 data, this discrepancy was more than 1000-fold if metagenomic sequences are also considered. The validation of AF2 by CASP14^42^ has given confidence in the quality of the AF2 structural models, and the release of 214 million AF2 models for UniProt represents a landmark in the study of protein structure and protein evolution.

One of the biggest immediate challenges is to handle the scale of the data since AlphaFold DB released a further 214 million model structures. This expansion in the data represents a 400-fold increase in the number of protein structures. Traditionally, structure comparison algorithms have been much slower than sequence comparison methods. However, the launch of AF2 occurred almost in parallel with the development and release of an extremely fast new comparison method, Foldseek^30^. Foldseek has comparable accuracy to the TM-align method^31^, traditionally used to assess structural similarity by many biologists and also employed by the CASP evaluation committee, but is 20,000 times faster. In addition, related machine learning tools to those employed by AF2, such as our CATHe predictor, that exploit language models to capture information about the structural contexts of residues, have become widespread and are being applied in the field of protein family classification as they are more powerful and faster than HMM based approaches^28,29,43–45^. These new technologies will harness the information from AF2 to enhance our understanding of fold space and the structural mechanisms by which structural changes impact on the functions of proteins.

In this work, we have built on classification workflows developed over the last 25 years for classifying protein domains in the CATH database^23^. These include strategies for identifying domain regions in protein sequences (CATH-HMM, CATH-Resolve-Hits^26^) and strategies for detecting very remote homologies by sensitive structure comparison methods (SSAP^32^). To handle the scale of the AF2 models, we built the CATH-Assign protocol, which used these approaches together with an extremely fast structure comparison method (Foldseek^30^) and also a novel protein language model (ProtT5^28^ developed by the Rost group) in a classifier (CATHe^29^) for detection of extremely remote homologues (<20% sequence identity) (see Fig. 1).

We have concentrated on identifying AlphaFold domains having characteristics similar to those of well packed globular domains in the Protein Data Bank. These domains could be classified in CATH because they could be matched to existing CATH domains by homology (CATH-HMM) or CATHe and validated by structure comparison. For the remaining putative domains we manually analysed a subset of human-containing families, in order to curate the domain boundaries and determine whether these were extremely remote homologues or were novel families.

We were able to process 369,512 confident AF2 models in less than six months. Since CATH-Assign is now established, new releases of AF2 models will be processed much faster. The near 70% expansion in structural data in CATH is impressive for such a short time scale. Prior to AF2, CATH contained nearly half a million experimental structures, in 5660 superfamilies. Classification of these superfamilies was performed over 25 years and benefited from substantial manual curation. Since coverage of CATH superfamilies by experimental structures is extremely sparse (on average <5%) structural validation can be difficult for very remote homologues. However, the release and classification of 214 million AF2 models for UniProt, will help curation of remote homologues as structural coverage of superfamilies will significantly increase. Furthermore, most of the AF2 domains brought in from the model organisms were very easy to process automatically as they had high structural similarity (SSAP score >85 out of 100) to structures already classified in CATH. The expanding structural coverage of superfamilies will also lead to an improvement in domain boundary detection for new AF2 models as they will be more likely to have close homologues in CATH superfamilies.

We remove nearly 50% of predicted domain models from our analyses, depending on the organism (see Supplementary Fig. 9). This is in agreement with early studies that calculated the percentage of disordered and low-quality regions in AF2 models in *Homo sapiens*^46^. The percentage of discarded domains is higher for Eukaryotes, with the exception of yeasts (baker’s and fission) (Supplementary Fig. 10). A large proportion (52%) of these have poor model quality (pLDDT < 70) and residues not predicted as ordered (i.e., not in secondary structures according to DSSP^47^) (3%). We also removed domains containing long continuous regions with no secondary structure, LURs, (>= 30% of the residues in the protein), as it would be difficult to match these domains to the more ordered protein domains in CATH (only 2.7% of CATH domains have LURs). It is not clear whether these regions are disordered or regions where AF2 struggles to model the conformation of the residues. They may also represent regions that undergo conformational change on binding to other proteins and, therefore capable of adopting multiple confirmations. We found quite a large number (79,825, 23%) of predicted domains with less than three secondary structures. From manual inspection, many comprise well-predicted alpha helices, which were often not packed against each other or domains in the proteins (see Fig. 7d for an example). It would be interesting to seek sequence relatives across diverse species for evidence of conserved residues suggesting functional roles.

Our results give an interesting perspective on the structural models in the 21 model organisms. CATH-Assign brought 92.3% of AF2 confident models (having no problematic features) into CATH superfamilies. Our study manually evaluated putative new superfamilies in human and identified 25 novel superfamilies. Although our results suggest another 2,367 new superfamilies in the other 20 organisms, as discussed many may match AF2 models pulled into CATH in the future. It is also likely that AF2 relatives pulled into CATH will include ‘bridging’ relatives that allow us to merge CATH superfamilies.

Taking into account the proportion of removed problematic models, CATH could expand by 150-fold or more once the new UniProt models are brought in over the next year. Development of CATH-Assign and the establishment of stringent and well benchmarked thresholds for HMM, CATHe, Foldseek, and SSAP matches puts CATH in a good position to process this data in a timely way. The expansion in structural coverage in CATH provided by curated sets of AlphaFold domains will be very helpful for functional and evolutionary studies, as well as protein domain boundary assignments^48^, and multi-domain structure modelling among other possible applications^49^. Work is ongoing to optimise our protocol to favour more prominently faster methods such Foldseek and TM-align and gradually bring in the entire release of UniProt, using a progressive approach that processes a batch at a time in the order of AF2 domains most similar to the domain structures classified in CATH.

We will continue to refine our characterisation of the novel superfamilies and exploit the expanded structural coverage of CATH superfamilies to further probe the relationships between structure and function. CATH-Assign will be applied to further releases of AF2 models. In addition, we are currently developing a deep-learning based approach for domain boundary assignment that combines homology data with deep-learning based features to improve boundary resolution for novel domain families.

The data is available grouped by CATH Superfamily and by organism through the 3D-Beacons network^25^, Zenodo (10.5281/zenodo.7404988, https://zenodo.org/record/7404988), and the CATH FTP (ftp://orengoftp.biochem.ucl.ac.uk/alphafold/cath-v4.3.0-model-organisms).

In summary, recent developments in deep learning methods applied to the analysis of protein structures (i.e., Foldseek) and protein sequence (i.e., pLMs exploited for classification - CATHe) have enabled the rapid processing of 708,941 predicted domain models generated by AF2. Although nearly 48% of domains from the model organisms were removed (because they were poorly modelled or had features that made them problematic for structure comparisons to globular domains in CATH), of the remaining domains 92% could be assigned to one of 3253 CATH superfamilies. We identified 3081 putative novel superfamilies. We manually examined a subset of 618 of these found in human and identified 25 which currently appear to be novel. The small number of new superfamilies identified to date is perhaps not surprising. In contrast, the expansion in structural diversity in CATH superfamilies (i.e., 36% increase in global fold groups) brought by AF2 relatives is exciting, as it could help rationalise functional divergence in these superfamilies.

## Methods

### 3D Models retrieval and processing

A total of 365,184 3D-models for 21 model organisms modelled with AlphaFold2 (AF2) were retrieved from the AlphaFold Protein Structure Database (v1) FTP (https://www.alphafold.ebi.ac.uk/)^12,14^. Due to a tool crashing on non-ATOM records, all models were stripped of all non-ATOM records. The sequences of the AF2 models are based on reference proteomes from UniProt, therefore MD5 hashes for each protein sequence in the reference proteomes were generated to facilitate tracking of pre-existing annotations, mapping of unique domain sequences and to avoid differences in naming across CATH, UniProt reference proteomes and AlphaFold DB structures.

### MD5 hashes and domains previously assigned by CATH-HMM and Pfam

For each unique MD5s in the dataset we assigned predicted protein domain boundaries. Gene3D assigns CATH domain annotations to UniProt entries by scanning their sequences against a library of 62,915 Hidden Markov Models (HMMs) seeded by a structural representative from each cluster of CATH relatives (at 95% sequence identity)^50^. Sequences are also scanned against a library of HMMs from structurally uncharacterised Pfam famililes.

Existing CATH and Pfam annotations and their boundaries were retrieved from Gene3D and used as input for CATH-Resolve-Hits (CRH)^26^. CATH-Resolve-Hits assigns the best possible combination of domains for a protein sequence to obtain the optimal coverage. A region in each protein could be therefore assigned to a CATH domain, a Pfam domain or to a domain-sized region unassigned to either of those (dubbed ‘NewFams’) (Fig. 1). In our protocol we used a 40-residues criterion to recognise domain sized regions as this threshold has been used to populate CATH.

### CATH superfamily predictions for Pfam and NewFams domains

We assigned a tentative, to-be-validated, superfamily CATH code for each domain sequence (Pfam and NewFams) using CATHe, a deep-learning based method for detecting remote homologues for CATH superfamilies. The first step in the CATHe pipeline was to convert the sequence domains already assigned to CATH superfamilies via matches to CATH-HMMs into a numerical representation (sequence embedding) using the ProtT5 protein Language Model (pLM). The pLM provides residue level embeddings which are then mean-pooled to obtain the embedding for the entire protein sequence. An Artificial Neural Network model was employed to learn from these sequence embeddings and to predict the superfamily annotations for new sequence domains. CATHe was trained on 1773 CATH superfamilies and attained a prediction accuracy of 85.6% (95% confidence interval) on them. The most highly populated CATH superfamilies are associated with a CATHe prediction accuracy of 98.2% (29). In order to use this model to make new predictions, we conducted a threshold analysis from which we concluded that a 40% prediction probability (correlating to an error rate of 5%) was optimal for our use case. Domain assignments below the 5% error rate threshold were marked as unassigned.

### Domain chopping from AlphaFold2 models

Predicted domains were chopped from the AF2 models using a built-for-purpose Python pipeline based on the pdb-selres module from pdb-tools^51^. The algorithm uses CATH-Resolve-Hits (CRH) output files or CATHe predictions and performs some initial checks, such as a lookup of the MD5 for the corresponding proteome and assigning the MD5 to a UniProt entry.

AF2 uses multiple models for large proteins over 2700 residues, providing 1400 amino acids long, overlapping fragments that are shifted by 200 residues. Based on the predicted domain boundaries, the algorithm detects which fragment contains the full domain, chops and creates a new PDB file with additional headers with metadata such as the domain MD5, the file from which it was chopped, and if available, the assigned Pfam family or predicted CATH superfamily.

### Chopped domain quality assessment

The quality of the domain was calculated as the average pLDDT^12^ of the constituent residues of the domain.

### Long Unordered Regions

Using pLDDT-per-residue scores, we identified Long Unordered Regions (LUR) as regions at least five residues long with a pLDDT < 70. Domains with more than 30% of residues in a single LUR were discarded.

### Secondary structure elements and order predictions

The secondary structure of each domain was assessed using DSSP^47^, with the resulting files optimised on secondary structure element lengths by secmake (https://github.com/UCLOrengoGroup/secmake). The DSSP predictions were used for secondary structure elements assignments and filtering. The overall unordered prediction was calculated as the percentage of residues not part of secondary structure elements over the total number residues. Domains with more than 65% of residues unordered were removed from our classification analysis.

### Packing density and globularity predictions

We used two metrics to predict the globularity of the domains obtained from the AF2 structures. The first one predicts the packing density by calculating the average number of neighbour residues each hydrophobic residue in the protein has within 5 Å. This was done using the python Bio.PDB package^52^. While this first metric considers the chemical aspect of the residue, the second metric predicts globularity on a mechanical level, by calculating the surface and volume of the domain. Using the programme MSMS included in PyMOL^53^, we obtained both the solvent excluded surface (SES) area as well as the volume resulting from it. For the metric, we then calculate the quotient of SES area/Volume. The more globular a protein is, the smaller this value should be.

To obtain thresholds for both metrics we ran them on the set of domain structures within CATH, which have been hand-curated over the years. There are a total of 61,238 domains in this dataset, which we constrained to only alpha, beta and mixed alpha-beta proteins. In order to account for errors in the dataset, we took as a globularity threshold the top 95% of hits in the dataset. This results in a packing density of 9.75, and a SES area/Volume value of 0.494 (Supplementary Fig. 3). Any domain with scores below these thresholds were discarded.

### Superfamily assignment validation protocol

We created a library comprising non-redundant representatives (at 95% sequence identity - S95s) for all 6331 superfamilies in CATH v4.3. We performed an initial scan of all query domains against the library of S95 representatives for the predicted superfamily using Foldseek, developed by the Steinegger Group (https://github.com/steineggerlab/foldseek), with a minimum overlap set at 0.4, coverage mode based on query and sensitivity set at 9. After extensive benchmarking (see Supplementary section Foldseek Benchmark, Supplementary Figs. 13 and 14), we set an overlap threshold of 60% and bitscore thresholds of 106 (for classes 1 and 3) and 165 (for class 2) for superfamily recognition. All queries with hits above the threshold were set aside, while the remainder were run against the same library using the in-house SSAP using established thresholds for homology (Supplementary section SSAP Benchmark, Supplementary Figs. 11 and  12)^32^.

### Clustering of unassigned domains into tentative new Superfamilies

All domains for which no superfamily could be assigned were scanned in an all-vs-all fashion using Foldseek with an overlap set at 60%, bitscore of 165 (the strictest for any given CATH class, see Supplementary section Foldseek Benchmarking) and sensitivity set at 9. The resulting output file was then fed into TCluster^54,55^ ran with single linkage clustering using the bitscores as weights for clusters cut-off.

### Diversity of positions

The Diversity of Positions (DOPS) score was calculated using the scorecons programme provided by the cathpy Python package^56^. The DOPS score, ranging from 0 (low diversity) to 100 (high diversity) considers the different conservation and frequency of residues across a multiple sequence alignment.

### Reporting summary

Further information on research design is available in the Nature Portfolio Reporting Summary linked to this article.

## Supplementary information

Supplementary Information

NR Reporting Summary

## Data Availability

The CATH-AlphaFold2 domains modelled and assigned with confidence are available grouped by CATH Superfamily and by organism on Zenodo (10.5281/zenodo.7404988, https://zenodo.org/record/7404988), and the CATH FTP server (ftp://orengoftp.biochem.ucl.ac.uk/alphafold/cath-v4.3.0-model-organisms). A table accompanying the results on Zenodo contains information for each confident CATH assignment on model quality, metrics, CATH SuperFamily assignment and its source. Individual models are available through the 3D-Beacons network^25^.

## Code availability

All tools and software used in these analyses and visualisations are peer-reviewed and freely available at the links provided in their article references, GitHub or PyPI. Foldseek is available via the Foldseek webserver (https://search.foldseek.com/) and GitHub (https://github.com/steineggerlab/foldseek). SSAP is available at https://cath-tools.readthedocs.io/en/latest/tools/cath-ssap/. pdb-tools is available through PyPI with instructions at https://www.bonvinlab.org/pdb-tools/. TM-align is available on the Zhang Group webserver at https://zhanggroup.org/TM-align/. DSSP is available at https://swift.cmbi.umcn.nl/gv/dssp/. Data was analysed using Python parsers and command-line scripts and visualised using the Seaborn Python package (https://seaborn.pydata.org/), Apple Keynote and Apple Numbers.
